# Low‐Grade Appendiceal Mucinous Neoplasm: A Case Highlighting Diagnostic and Management Considerations

**DOI:** 10.1155/carm/7538312

**Published:** 2026-01-27

**Authors:** Ali Hamdan, Jana Khalil, Razan Abou Zeid, Kelly Katherine Karam, Philippe Attieh, Karam Karam, Lamia Azizi, Elias Fiani

**Affiliations:** ^1^ Department of Internal Medicine, University of Balamand, Beirut, Lebanon, balamand.edu.lb; ^2^ Department of General Surgery, University of Balamand, Beirut, Lebanon, balamand.edu.lb; ^3^ Department of Gastroenterology, University of Balamand, Beirut, Lebanon, balamand.edu.lb; ^4^ Department of Radiology, University of Balamand, Beirut, Lebanon, balamand.edu.lb

**Keywords:** appendectomy, appendiceal mucoceles, low-grade appendiceal mucinous neoplasms (LAMNs)

## Abstract

Appendiceal mucoceles (AMs) are rare pathological entities characterized by the accumulation of mucin within the appendiceal lumen. They may arise from either non‐neoplastic or neoplastic processes, including low‐grade appendiceal mucinous neoplasms (LAMNs). Due to their variable clinical presentation and often incidental discovery, diagnosis can be challenging. We report the case of a 29‐year‐old female who presented with a 3‐month history of constipation and intermittent abdominal pain. Physical examination revealed mild right lower quadrant tenderness without signs of peritonitis. Laboratory findings were unremarkable. Abdominal CT demonstrated a cystic, fluid‐filled structure at the base of the appendix. Colonoscopy revealed a glossy, dome‐shaped protrusion at the appendiceal orifice, consistent with a mucocele. The patient underwent an elective laparoscopic appendectomy. Histopathologic examination confirmed the diagnosis of LAMN. Her postoperative course was uneventful, and she remained asymptomatic on follow‐up. This case illustrates the diagnostic complexity of appendiceal mucoceles, particularly in young adults, where the condition is uncommon. The combination of cross‐sectional imaging and endoscopic evaluation was pivotal in establishing the diagnosis. Early surgical management was crucial to prevent rupture and the potential development of pseudomyxoma peritonei. Clinicians should maintain a high index of suspicion for AMs in patients with unexplained gastrointestinal symptoms and characteristic imaging findings. Prompt diagnosis and appropriate surgical intervention ensure favorable outcomes and prevent serious complications.

## 1. Introduction

Appendiceal mucoceles (AMs) are rare entities characterized by a distended, mucous‐filled appendix [1]. The incidence of appendiceal mucocele is 0.2%–0.7% among appendectomy samples [2]. This disease can result from benign or malignant pathologies: retention cysts, cystadenomas, cystadenocarcinomas, and mucosal hyperplasia [3]. Mucocele of the appendix has no specific clinical features [4]. AMs are incidentally found while undergoing radiologic or endoscopic procedures or pathologic specimens following appendectomy. Hence, the diagnosis of appendiceal mucocele is challenging, requiring a combination of imaging, endoscopic, and histopathological techniques for an exact identification and treatment [5]. Timely diagnosis and appropriate surgical management are essential to prevent spontaneous rupture and subsequent seeding of mucin cells into the peritoneal cavity. Histopathologic analysis of the excised specimen identifies the nature of the mucocele and further guides management. This case report highlights the importance of combining diagnostic procedures to identify appendiceal mucocele in patients presenting with nonspecific symptoms.

## 2. Case Presentation

A 29‐year‐old female, with no significant medical history, presented with a 3‐month history of constipation despite daily use of Metamucil (psyllium fiber), accompanied by intermittent abdominal pain and bloating. The patient’s vital signs on presentation were stable.

Abdominal physical examination revealed mild tenderness in the right lower quadrant without rebound tenderness or guarding. Investigations including complete blood count and basic metabolic panel were within normal limits.

A contrast‐enhanced computed tomography (CT) scan of the abdomen and pelvis was performed, revealing a nonenhancing, fluid‐filled structure predominantly at the base of the appendix, without surrounding fat stranding or evidence of acute inflammation (Figure 1).

**Figure FIGURE 1:**
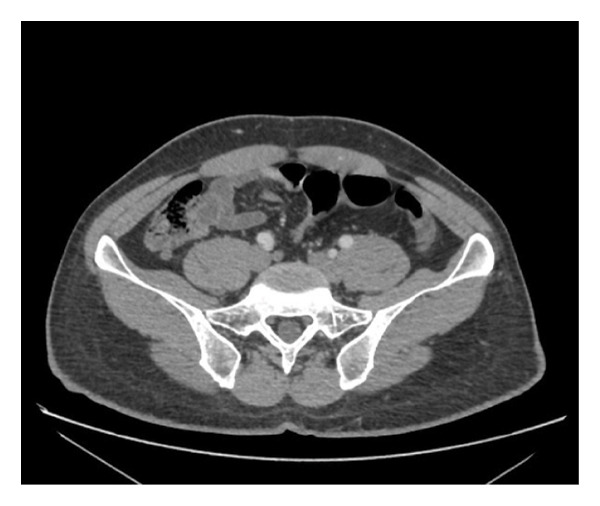
A contrast‐enhanced CT scan of the abdomen and pelvis revealing a nonaerated, fluid‐filled structure predominantly at the base of the appendix, without surrounding fat stranding or evidence of acute inflammation.

Subsequently, the patient underwent colonoscopy, which identified a white, glossy, roundish structure protruding out of the lumen of the appendix, suggestive of a mucocele (Figure 2). A biopsy was not performed during colonoscopy, as obtaining tissue from a suspected appendiceal mucocele or LAMN carries a risk of mucin leakage and peritoneal dissemination. Given these findings, the patient underwent elective laparoscopic resection of the appendiceal mass. Intraoperatively, the appendix appeared grossly enlarged and filled with mucoid material. Pathological examination of the resected specimen showed a 6 × 1 cm appendix containing bloody purulent material (Figure 3). Histological features included low‐grade epithelial atypia, enlarged pseudostratified nuclei, abundant cytoplasmic mucin, mural fibrosis without destructive invasion, and absence of complex architectural features, which were consistent with low‐grade appendiceal mucinous neoplasm (LAMN) according to the 2019 World Health Association (WHO) and Peritoneal Surface Oncology Group International (PSOGI) classification criteria. The postoperative course was uneventful, and the patient was discharged home on the second day of surgery. She has been asymptomatic on every follow‐up visit.

**Figure FIGURE 2:**
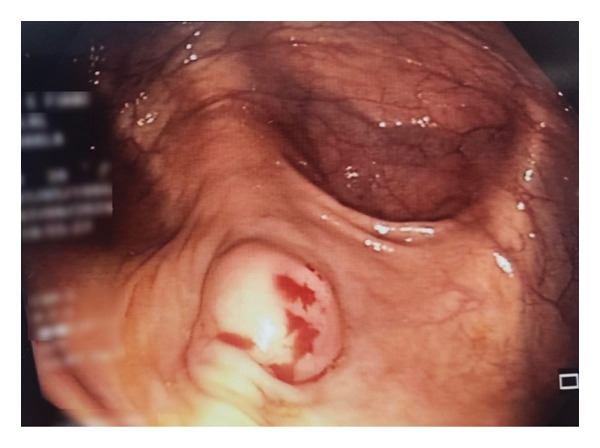
Another colonoscopic view identifying a white, glossy, roundish structure protruding out of the lumen of the appendix, suggestive of a mucocele.

**Figure FIGURE 3:**
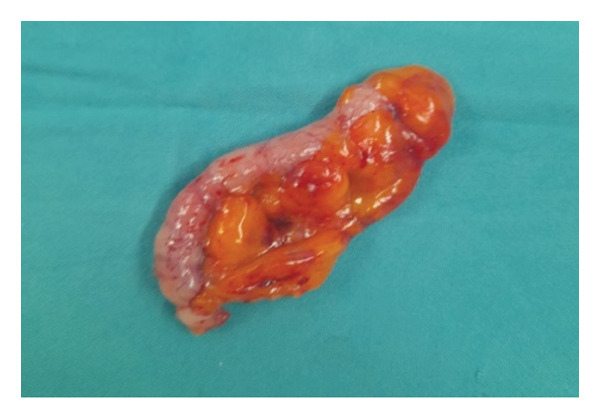
Grossly enlarged 6 × 1 cm appendix filled with bloody purulent material.

## 3. Discussion

AMs have a female predominance, mainly occurring in the fifth to sixth decade of life [6]. Nevertheless, our case involved an appendiceal mucocele at the age of 29, demonstrating that it can occur in younger age groups. AMs are generally classified into four different histological subtypes as retention cyst, mucous hyperplasia, mucinous cystadenoma, or mucinous cystadenocarcinoma [7]. However, appropriate categorization and nomenclature of these neoplasms remains problematic, with various pathologic grading systems [5]. When evaluating the PSOGI and WHO classification systems altogether, appendiceal non‐neuroendocrine epithelial tumors can be grouped into several categories: serrated polyps with or without dysplasia, LAMN (low‐grade atypia), high‐grade appendiceal neoplasm (HAMN) (high‐grade atypia), mucinous adenocarcinoma (varying differentiation and mucin content), poorly‐differentiated adenocarcinoma with signet ring cells, mucinous signet cell adenocarcinoma, nonmucinous adenocarcinoma (resembling colorectal type), and goblet cell adenocarcinoma [7].

Histopathologic examination of our patient’s specimen confirmed the diagnosis of LAMN.

Patients with LAMN often present with nonspecific symptoms, the most common being abdominal pain, but other reported symptoms include palpable abdominal mass, weight loss, and changes in intestinal function or genitourinary symptoms [5, 7, 8]. However, some patients remain asymptomatic, with the mucocele discovered incidentally during evaluation of other conditions. Our patient presented with constipation unresponsive to fiber supplementation, accompanied by intermittent abdominal pain and bloating, demonstrating the nonspecific features of the disease. The rarity of the disease and the nonspecific nature of its clinical symptoms pose diagnostic challenges in identifying LAMN.

Preoperative diagnosis is crucial for the determination of the surgical approach and its extension [8]. Multiple imaging modalities can be used to evaluate LAMN including CT, ultrasonography (US), or magnetic resonance imaging (MRI) [3]. An important sonographic parameter that can help differentiate LAMN from acute appendicitis (AA), given their overlapping symptoms, is the outer appendiceal diameter. Onion skin appearance and an outer diameter of 15 mm or more should prompt consideration of LAMN [4, 5]. However, CT imaging is considered a superior modality and is more commonly utilized to establish an initial diagnosis [3]. CT can effectively show well‐circumscribed cystic masses with mural calcifications, which are important diagnostic clues. It can also depict the luminal diameter and the anatomical relationship between the elongated cystic mass and the cecum [8]. Imaging clues suggestive of malignancy include mural nodularity and irregular wall thickening [9]. It is crucial to detect the presence of extra luminal mucin, manifesting as low attenuation nodules containing coarse calcification, to which MRI offers higher sensitivity for detection than CT [9]. Colonoscopy can be used to evaluate other colonic lesions and diagnose synchronous or metachronous colonic cancers [2]. A pathognomonic sign of LAMN, as was seen during colonoscopy evaluation in our patient, is the “volcano sign,” which appears as a mount‐like elevation of the appendiceal orifice with an erythematous, soft mass and a central crater discharging yellowish mucus [8, 9].

Timely diagnosis and appropriate surgical approach are essential in order to prevent spontaneous rupture and subsequent seeding of mucin cells into the peritoneal cavity. This feared complication of appendiceal mucinous neoplasms is known as pseudomyxoma peritonei (PMP), which results in a worse prognosis [5]. In general, open laparotomy is favored over a laparoscopic approach to ensure intact removal without the risk of seeding and to facilitate exploration for colonic and ovarian tumors, given their association with LAMN [6, 8]. This also allows assessment of appendiceal lymph nodes and margins and inspection for potential mucinous fluid accumulation in the pelvis, paracolic gutters, greater omentum, and perihepatic spaces [6, 8]. A simple appendectomy is recommended for benign mucoceles with a normal cecum and appendicular base with no evidence of perforation [6]. Notably, careful laparoscopic handling, as provided for our patient, may be acceptable if the mucocele appears to be a homogenous cyst with no nodularity and no signs of perforation [8].

LAMNs are dysplastic lesions with low cellularity, minimal mitotic activity, no signet cells, no angiolymphatic invasion, nor perineural invasion, with possible absence of the lamina propria and muscularis mucosae with no infiltration or invasion by malignant cells. LAMNs are therefore graded as well‐differentiated or G1 [10]. Confined LAMNs can be entirely removed through appendicectomy, and an additional right hemicolectomy is generally unnecessary. The approach to managing microscopically positive margins (R1) in unruptured LAMNs remains a topic of debate. The 2020 Chicago Consensus suggests that surgery should be completed with a cecectomy or ileocecectomy [11]. On the other hand, an observational follow‐up is also a valid choice for this patient group. However, the majority of experts advise against performing a right colectomy for confined R1 LAMNs [11].

Likewise, HAMN limited to the appendix is managed with appendectomy, but a comprehensive histologic evaluation is necessary to rule out invasive adenocarcinoma. Referral of patients with peritoneal mucin at initial surgery to specialized centers is essential [7]. For cases presenting with mucinous carcinoma or PMP, cytoreductive surgery combined with intraperitoneal chemotherapy offers a targeted treatment approach [8]. When a malignant mucocele is suspected by the presence of a perforated mucocele, an enlarged mesenteric lymph node, or a positive cytology, right hemicolectomy is advised [6].

Although cystic appendiceal lesions include both benign and malignant entities, cross‐sectional imaging in this patient demonstrated a well‐circumscribed, fluid‐filled appendix without mural nodularity, surrounding fat stranding, or lymphadenopathy. Although colonoscopy demonstrated elevation at the appendiceal orifice, suggesting possible involvement of the base, both preoperative imaging and intraoperative evaluation confirmed that the lesion was confined to the appendix without cecal invasion and no evidence of perforation. Based on these findings, laparoscopic appendectomy was deemed appropriate in accordance with current recommendations, which reserve ileocecal resection with lymphadenectomy for cases with suspected invasive malignancy or nodal involvement. Definitive diagnosis was subsequently achieved through histopathologic evaluation, confirming LAMN.

Although most AMs are related to obstructive or neoplastic mucosal processes and the WHO/PSOGI classifications address the neoplastic spectrum, appendiceal endometriosis represents an important non‐neoplastic mimic, especially in young women. Endometriotic implants involving the appendiceal wall have been documented in multiple series and case reports and, through serosal or mural involvement, may produce luminal obstruction and secondary mucocele formation [12, 13]. Prevalence estimates vary with the population studied (higher among women with known pelvic endometriosis or deep infiltrating disease), and appendiceal endometriosis is frequently an intraoperative or histopathologic diagnosis because preoperative imaging and endoscopy seldom distinguish it from other appendiceal lesions. Therefore, when discussing AM in younger patients, clinicians should include appendiceal endometriosis in the differential diagnosis and ensure appropriate histologic assessment of the resected specimen [12].

This case highlights the importance of considering LAMN in the differential diagnosis of young patients presenting with nonspecific gastrointestinal symptoms when coupled with suggestive imaging findings to avoid misdiagnosis and prevent complications. Although LAMNs are generally slow‐growing lesions with a relatively low rate of progression to PMP (estimated at ∼5%), elective surgical resection remains the standard of care once the diagnosis is established. Histopathologic analysis of the excised specimen identifies the nature of the mucocele and further guides management. Although appendectomy with negative margins is typically curative for LAMN confined to the appendix, periodic follow‐up is recommended as a precaution to ensure no delayed peritoneal mucinous disease develops, particularly in cases where occult microscopic mucin leakage cannot be fully excluded. In our case, a timely laparoscopic appendectomy facilitated safe and intact removal of the lesion without evidence of perforation.

## Author Contributions

All authors have contributed to this manuscript in terms of planning, conception, writing, and editing various drafts of the manuscript.

## Funding

No funding was received for this research.

## Disclosure

All authors have read and approved the final manuscript.

## Ethics Statement

The authors have nothing to report.

## Consent

Written informed consent was obtained from the patient for publication of this case report and any accompanying images. A copy of the written consent is available for review by the Editor‐in‐Chief of this journal.

## Conflicts of Interest

The authors declare no conflicts of interest.

## Data Availability

Data will be made available upon request from the authors.
